# Decoding the Biobased Blueprint: Key Players and Evolutionary Trends in Materials Innovation

**DOI:** 10.3390/polym17020177

**Published:** 2025-01-13

**Authors:** Silvia Rita Sedita, Eleonora Di Maria, Leonardo Mazzoni, Negalegn Alemu Bekele

**Affiliations:** 1 Department of Economics and Management, University of Padova, 35123 Padova, Italy; eleonora.dimaria@unipd.it; 2 European University Institute, Robert Schuman Centre, Centre for a Digital Society, 50133 Florence, Italy; leonardo.mazzoni@eui.eu; 3 Department of Mechanical Engineering, Debre Markos University, Debre Markos 251, Ethiopia; negalegnalemu.bekele@unipd.it

**Keywords:** innovation landscape, biobased materials, BIOMAC, technological leadership, freedom-to-operate, exploitation, commercialization, patent analysis

## Abstract

In the rapidly evolving biobased materials innovation landscape, our research identifies key players and explores the evolutionary perspective of biobased innovation, offering insights into promising research areas to be further developed by biobased material scientists in search of exploiting their knowledge in novel applications. Despite the crucial role of these materials in promoting sustainable production and consumption models, systematic studies on the current innovation terrain are lacking, leaving gaps in understanding key players, emerging technologies, and market trends. To address this void, we focused on examining patents related to biobased monomers and polymers, aiming to describe the innovation strategies and business dynamics of leading assignees. Embedded within the European Sustainable BIO-based nanoMAterials Community (BIOMAC) project, a Horizon 2020 initiative, our research leverages this unique framework dedicated to advancing the innovation landscape, specifically emphasizing the market readiness of biobased materials. We implemented a multi-stage strategy, prioritizing validated keyword queries to ensure the superior quality and reliability of the collected data. To understand primary contributors within these landscapes, we conducted an in-depth analysis of innovation strategies employed by leading companies. Findings from the ORBIT platform highlighted a remarkable increase in patent publications in the past decade, with China standing out as a key hub of innovation, signaling a strong focus on the development of these materials. Our research explores technological advancements in biobased materials to identify specific areas with potential for further development. By analyzing innovation trends in five key industries, we pinpoint opportunities for innovative solutions to be commercially exploited while ensuring compliance with intellectual property rights within a freedom-to-operate framework.

## 1. Introduction

Over the last decades, the concern of sustainability has emerged at the top of the policy agenda across many national and international constituencies, leading scientists to converge towards a sustainability-driven research agenda. One of the most lively fields of research is that of biobased materials, where researchers are challenged to explore novel solutions based on polymers derived from renewable biological resources (‘biobased’) and polymers that are considered ‘biodegradable’ [1]. Research in this field is promising because it offers solutions that allow the implementation of circular economy principles [2,3,4] both from the demand and the supply side, ultimately rethinking production and consumption processes to embrace the sustainability imperative. An increasing variety of biobased materials are now being developed for different applications such as packaging, agriculture, automotive, construction, and medical. The research in this area is inherently connected with the potential for market applications, asking for an extensive network of collaborations between academic and corporate researchers operating in diverse fields within a multidisciplinary and transdisciplinary perspective. The range of applications is often country-specific, with the effect of generating a technological space that is geographically unevenly distributed [5,6].

Innovation management studies so far somewhat overlooked advancements in biobased innovation [7]. Despite the crucial role of these materials in promoting sustainable production and consumption models, systematic studies on the current innovation terrain are lacking, leaving gaps in understanding key players, emerging technologies, and market trends. These facts call for an accurate examination of the technological landscape of biobased innovation, which can be performed by looking at the technological trajectories of biobased patents.

The objective of this study is to examine patents related to biobased materials, and especially the innovation space in biopolymers, and their potential to support the sustainability transition of the economy, also identifying the main companies actively involved in such innovation dynamics to be highlighted as key players in this field. By carrying this out, our work not only offers a comprehensive picture of the biobased innovation landscape from the industrial (supply) point of view but also increases the current awareness of the freedom to operate in biobased materials research [8]. Armed with this understanding, biobased material scientists can not only better evaluate the direction and commercial impact of their research efforts but also plan their collaboration networks accordingly.

Our research departs from the BIOMAC (European Sustainable BIO-based nanoMAterials Community) project, a Horizon 2020 initiative whose objective is to establish an Open Innovation Test Bed (OITB), a collaborative ecosystem where technologies and solutions utilizing nano-enabled biobased materials (NBMs) will be upscaled and prepared for market applications.

The empirical analysis focuses on the exploration of five test cases, which have been identified by BIOMAC experts as the most relevant to examine the applicability of biobased nanomaterials: automotive, agriculture, construction, food packaging, and printed electronics.

This work empirically analyses these five sectors to identify the boundaries of the biobased innovation landscape and to spot key players and their evolution over time through patent data analysis [9]. To summarize our results, the US and China are the leading countries in biobased innovation, with different roles from other countries (UK, Germany, Japan, and Korea) with respect to different domains of applications. According to our trend analysis, recent innovation trajectories are towards polymers, molecular chemistry, biotechnology, medical technologies, and textile applications. Key players with respect to patenting processes are Procter and Gamble, UPM, Arkema, Stora Enso, CNRS, BASF, and Novamont.

Our study examined the available patent data to show how companies are actively working towards biobased innovation and understand the market influence of such actively engaging innovation players. This is a unique way of contributing to the biobased materials innovation community to have an understanding of trends, trajectories, commercial impacts, and future potential of such innovations in the selected five applications.

The rest of this paper is organized as follows. Section 2 explains the conceptual backgrounds related to biobased innovation, patents, and key players. Section 3 illustrates the research methodology and sector specificity for the five test cases. Section 4 presents and discusses the findings. Section 5 advances some concluding remarks.

## 2. Background of the Research

### 2.1. Bioplastics Innovation Overview

The production of biobased plastics and the incorporation of biobased materials and their derivatives as monomers into conventional plastics have significantly expanded the content of natural products in polymer materials [10]. The development of biobased materials such as biodegradable polymers is recognized as one of the most successful innovations in the polymer industry to address environmental issues [11]. Such technology and product innovation have a crucial effect on market potential [12] and even more when it comes to responsible innovation, such as that of biobased materials. Moreover, it is possible to map technology evolution, which is often intertwined with industry evolution, because of some path-dependent attributes, i.e., the innovation path is not independent of previous significant evolutions but rather built upon the knowledge from previous innovations in a cumulative way [9]. The technological evolution of innovation in biobased materials is driven by the need to create more sustainable products. Biopolymers, bio-composites, biobased additives and fillers, and biodegradable and compostable materials are the most recent developments in this field. These materials have the potential to transform industries, reduce environmental footprint of production processes, and create new opportunities for sustainable development.

According to research such as [13,14], there are four different paths of innovation in the bioeconomy: (i) substitution of fossil fuels with biobased raw materials; (ii) boosting primary sector productivity; (iii) new and more efficient biomass uses, and (iv) low bulk and high-value application. The first path describes innovation that prioritizes replacing current items without changing their functionality, whereas the second path is about technological innovation that increases productivity in specific sectors. The third path is about innovations that increase the efficiency of biomass use and waste recycling. The last path is about innovations that provide superior functionality where the substitution of fossil fuel to biomass plays a minor role. However, cross-cutting bioeconomy perspective offers a useful lens for stakeholders (including innovators) in envisioning alternative pathways to sustainable futures and identifying tangible approaches for implementation [15].

Collaborative R&Ds and open innovation strategies [16] have been employed in developing such innovations for different applications. In achieving sustainable innovation of bioplastics, stringent environmental policies and perceived regulatory shortcomings can act as barriers to national specialization in biobased technologies [7], giving rise to a diversified regional scenario.

### 2.2. Measuring Biobased Innovation Through Patents

Patents, in general, are the primary source of invention and technology evidence [17,18]. A patent is a legal title granting its holder the right to prevent third parties from commercially using an invention without authorization [19]. They are a direct outcome of the inventive process and, more specifically, of those inventions that are expected to have a commercial impact [20]. They help survey innovative efforts and explore technological trajectories, the geographical distribution of the innovative effort, and the freedom to operate [18]. Technological space mapping through patents also allows the analysis of the knowledge recombination process that is usually at the basis of novelties, which are the result of past knowledge accumulation and new paths exploration (M.-C. Hu, 2008) [21]. Knowledge accumulation (stock of patents) [22] may improve inventive efforts and decrease inventive uncertainties [23]. The combinative capability of firms to generate new applications from the existing knowledge is responsible for the detection of novel technological opportunities [24].

Patent analysis has been conducted by several authors for different reasons. For example, to examine the emerging actors and collaborative networks in the nanotechnology innovation [25], to map firms’ locations in technological space [26], for the identification of the previous innovation effort (i.e., patented technologies within the stock of knowledge), ref. [27] highlighted the importance of using granted documents inside the patent portfolio.

Different research has been developed to measure the throughput of innovation in a biobased economy. Authors such as [13] used patents as indicators to evaluate innovation dynamics in Germany and outline constraints for commercial scenarios. To study the technological specialization of countries in biobased technologies, authors such as in [7] applied patent data. Authors in [13] also used patents and publications as indicators to examine bioeconomy transitions with economic, environmental, and innovation indicators. Authors in [28] performed patent landscape analysis to understand the growth of biobased (biosolution) production, where they found that the growth of biomass production-based biosolution is remarkably significant. However, patents are territorial and time-limited, and the possibility of discovering new market opportunities depends on the identification of unexplored (or less explored) arenas. The patent system not only serves to encourage and protect innovation but also injects stability and predictability into the market economy [29] thanks to knowledge disclosure. As an innovation activity, patent commercialization has been not only a business event but also a macro-social and economic event [30].

### 2.3. Key Players in the Biobased Innovation Landscape

Even though the consumption and production of biobased products such as biopolymers are low compared to their traditional counterpart, there are several successful examples of industrialization of these polymers, including pilot-scale production of polylactide (PLA) at NatureWorks and Corbion/Total; poly(trimethylene terephthalate) (PTT) at DuPont; poly(isosorbide carbonate) at Mitsubishi Chemicals (Chiyoda City, Japan); biobased polyamides at Arkema, Toray, BASF, DSM, and others; and poly(ethylene 2,5-furandicarboxylate) (PEF) at Synvina [11]. Research at the regional and firm/industry level is important to understand key players and innovation trends.

Geographically, the analysis at the country level shows that China’s innovation economy is rapidly becoming the largest in the world, and it is increasingly placing Intellectual Property (IP) rights at the center of systemic innovation attention, bridging innovation to social values [31]. China has a system of incentives to support systemic innovation, reflected in the increased number of IP applications and grants [5]. From 2000 to 2020, Asian and North American countries submitted significantly more non-patentable IP rights applications, but they were also more successful in obtaining IP rights [5]. According to the European Patent Office (EPO) report (2023), the top five countries of origin for filings in 2023 were the United States (+0.4%), which accounts for almost a quarter of all applications, followed by Germany (+1.4%), Japan (−0.3%), China (+8.8%) and new top five entrant R. Korea (+21.0%). For sustainability-related innovations, according to the World Intellectual Property organization (WIPO) report (2023), China’s exponential increase in patents becomes evident, moving from being at the bottom of the five selected countries/regions to very nearly second place in 2023. There have been noticeable increases from all areas, with Japan’s showing a modest increase and US growth almost matching that of China. The share of Sustainable Development Goal (SDG)-related patents is quite consistent across authorities, ranging from 34% to 38% in 2023. Europe shows the fastest rate of growth, whereas for China the increase remains below average compared to other regions.

At the firm and industry level, the scenario is mixed. Authors such as [8] examined the top 20 companies who filed patents in the last 20 years and identified that activity from universities (Univ. of Washington) and companies in the fields of diapers and single-use textiles (P&G, Kimberly Clark), therapeutic fields (Abbott, Obalon); packaging (Biotec); and material producers (BASF, Eastman, Metabolix (now CheilJedang), Kaneka, Roquette) are the leading ones. Recently, the biobased materials market such as the PLA market has witnessed increased demand over past years for various packaging applications (dry products and perishable products such as fruits and vegetables), resulting in increased production in Europe, the United States, and Japan with key players include Futerro (Belgium), NatureWorks LLC (MN, USA), BASF SE (Germany), Total Corbion (The Netherlands), Hitachi Ltd. (Japan), Sulzer Ltd. (Switzerland), Zhejiang Hisun Biomaterials Co., Ltd. (China), and ThyssenkruppAG (Germany) [32].

According to Market and Markets 2024 report, NatureWorks LLC (US), Braskem (Brazil), BASF SE (Germany), TotalEnergies Corbion (Netherlands), Versailles SPA (Italy), Biome Bioplastics Limited (UK), and Mitsubishi Chemical Group (Tokyo, Japan) are the big players in biopolymer market currently. These companies mainly focused on biobased (biodegradable or non-biodegradable) material innovations for applications in packaging, consumer goods, automotive and transportation, textiles, coatings and adhesives, building and construction, and electrical and electronics sectors. The report by Verified Market Research in 2023 also identified that the major players in the biobased market are BASF SE, Archer Daniels Midland Company, Bio-on S.p.A., Braskem S.A., Danimer Scientific, Du Pont, NatureWorks LLC, Novamont S.p.A., Plantic Technologies Ltd. and Rodenburg Biopolymers B.V. Packaging, healthcare, agriculture, consumer good, automotive, textile, and construction are the major applications for such innovations. The European bioplastics 2023 report also highlighted that the main applications of the bioplastic innovations are packaging (43%), fibers (21%), consumer goods (13%), automotive and transport (10%), agriculture (5%), electronics (4%), and others (5%).

In sum, players such as Novamont, NatureWorks, Bio-on, Mitsubishi Chemicals, BASF, Braskem, Total Corbion, and DSM are working on biobased innovations for applications in food packaging, compostable materials, textile, electronics, agriculture, water-resistant coatings, biomedical applications, automotive, and consumer goods [33].

Despite the availability of general information about these companies and application areas, which players are playing significantly in which areas of commercial applications still need further investigation to better outline the technological trajectories and innovation paths at the firm and sector levels.

## 3. Research Methodology

### 3.1. Research Design

**Figure 1:**
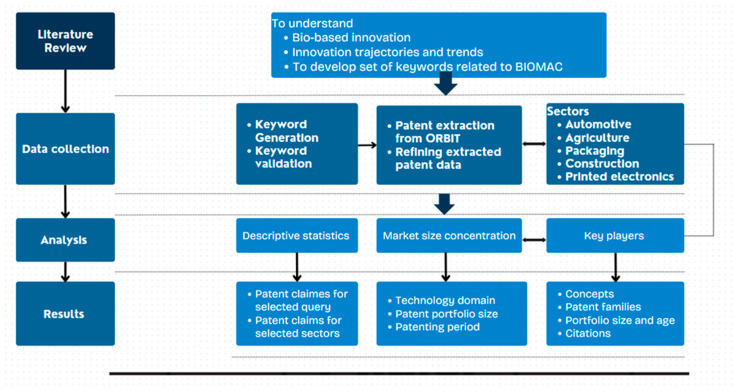
Research framework.

This research mainly depends on data extracted from the ORBIT database. We first identified a set of keywords related to biobased innovation, which was useful for retrieving data from the ORBIT database, and second, we conducted an in-depth analysis of the five test cases mentioned above (The five test cases are automotive, agriculture, food packaging, construction, and printed electronics. The choice of the sectors derives from the recommendations of BIOMAC technical experts since they are considered the most relevant to examine the exploitation of biobased nanomaterials). The research framework is illustrated in Figure 1.

### 3.2. Data Collection

**Figure 2:**
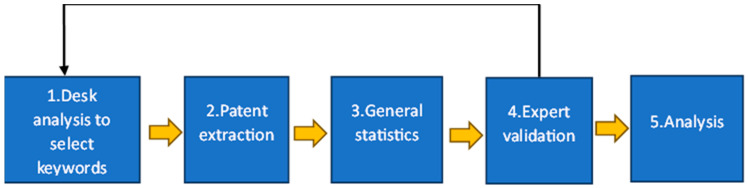
Patent search and analysis strategy.

We built an original database on biobased materials patents from the ORBIT platform. ORBIT is a cloud-based service dedicated to IP search and allows multiple searches across national and regional patent authorities, including the IP that is available worldwide. It gives the possibility to collect and analyze information on IP. In order to obtain a high-quality database that takes into account multi-domain technology, such as the combination of nanomaterial and biobased polymers, we opted for a multi-stage strategy. Sample selection relying solely on the technological classes (such as International Patent Classification (IPC) and Cooperative Patent Classification (CPC)) risks, in fact, to under (or over) estimate the patenting landscape. Moreover, we also considered the fact that the use of synonyms, acronyms, and molecular formulas is common in chemistry (e.g., polyethylene has various synonyms such as ethylene homopolymer, ethylene polymer, polythene, or acronym such as “PE”), with the consequence of identifying the same term with multiple keywords. Considering this premise, this paper adopts the following multi-stage strategy (Figure 2), which is reiterated until receiving final approval from BIOMAC experts.

I. Desk analysis of the technical literature to identify a set of keywords related to the biobased materials.
II. Extraction of patents according to the set of keywords (including combinations of them) using Boolean operators and truncations.
III. General statistics (the total number of patents, the main assignees, the technological classes involved, keywords accuracy).
IV. Validation of the selected keywords will be done by meetings with technical experts in the BIOMAC project.

The adoption of a sequential approach, composed of a first phase of autonomous research (the first 3 steps) and a second phase of validation, improved the accuracy of the final database, a very delicate phase of IP analysis, especially when emerging technological domains are treated.

Once the database has been built, we first described the technological landscape. The goal of the analysis is to identify market leaders and main technological areas in terms of the number of patents, temporal trends, and domains. For this purpose, we collected and analyzed the following information:

- Identification numbers (Application number; Publication number(s); Unique ID Questel);
- Dates (Application date; Publication date(s); Grant date; Expected expiry dates);
- Biblio ((Title; Abstract; Assignee (name, address, country); Inventor(s) (name, address, country); Technical concept(s));
- Claims;
- Classification (Technology domains; IPC; CPC);
- Key content (Advantages/Previous drawbacks; Object of the invention);
- Citations ((Backward citations (number and patents ID); Forward citations (number and patents ID)).

To select keywords, a wide family of materials (biodegradable and non-biodegradable) that are included in the scope of BIOMAC have been considered. This includes biobased chemicals/monomers (such as Succinic Acid, Lactic acid, Sugar alcohols/polyols, and diols/glycols), Bioplastic (such as PLA; biobased polyester and polyurethane resins; Biochar), and biobased nanomaterials (such as Nanolignin; Nanocellulose). Recent reviews of the literature and the European Bioplastics Association also provide a broader range of keywords to be tested in the search strategy. For instance, the European Bioplastic Association reports three groups of bioplastics:

(1) Biobased (or partly biobased), non-biodegradable plastics, such as biobased polyethylene (PE), polypropylene (PP), polyethylene terephthalate (PET) (so-called drop-in solutions), biobased technical performance polymers, such as numerous polyamides (PA) or polytrimethylene terephthalate (PTT).
(2) Biobased and biodegradable plastics, such as polylactic acid (PLA), polyhydroxyalkanoates (PHA), polybutylene succinate (PBS), and starch blends;
(3) Plastics that are biodegradable and based on fossil resources, such as polybutylene adipate terephthalate (PBAT), may well be produced at least partly biobased in the future.

**Figure 3:**
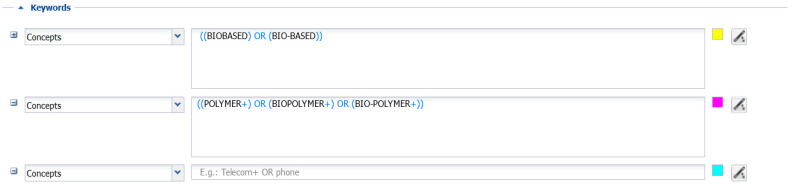
Illustration of setting keywords with ‘+’ sign in ORBIT.

**Figure 4:**
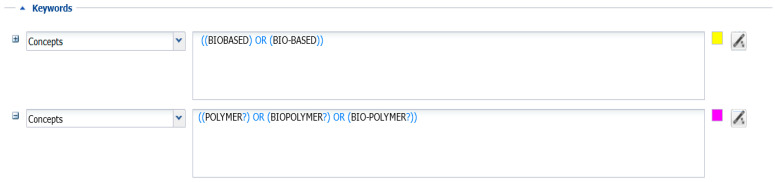
Illustration of setting keywords with ‘?’ sign in ORBIT.

A similar tree-based classification with the employment of further keywords and acronyms is reported by [34]. From such frameworks emerge some common set of keywords and acronyms. Moreover, a sound use of operators and truncations is also requested, given the many possibilities offered by ORBIT. From the suggested operators, changing only the ‘+’ sign with ‘?’ reported different results, as shown in Figure 3 and Figure 4.

(((BIOBASED) OR (BIO-BASED))/KEYW/TI/AB AND (((POLYMER+) OR (BIOPOLYMER+) OR (BIO-POLYMER+)))/KEYW/TI/AB)

Results = 8003 unique patent families.

(((BIOBASED) OR (BIO-BASED))/KEYW/TI/AB AND (((POLYMER?) OR (BIOPOLYMER?) OR (BIO-POLYMER?)))/KEYW/TI/AB)

Results = 6796 unique patent families.

In the final stage, we carried out 20 queries for materials and 27 for test cases. We employed the global landscape, US, and European Union searches.

In general terms, this strategy has brought satisfactory results in terms of the capacity of European patents to represent the global coverage of materials search (26% on average for biobased monomers and 30% for biobased plastic); there are few exceptions concerning nanolignin, biochar, and nanocellulose in the macro theme of biobased nanomaterials (these are below 10%). Concerning test cases, polyurethane construction and nanocomposite construction are the only two queries largely underrepresented in the sample (below 10%). Concerning the typology of the query, we test four options (concepts; title and abstract; title, abstract and claims; title, abstract, claims and object of the invention). We decided to rely on the last one as it guarantees the best equilibrium between precision and the number of results among the four options.

### 3.3. Test Cases: Sectoral Specificities and Topic Analysis

This work provides an in-depth analysis of five test cases: automotive, agriculture, construction, food packaging, and printed electronics. These cases were selected by the BIOMAC project as the most relevant and promising for the commercialization of biobased innovation. The first is the automotive industry, a sector under huge pressure by markets and regulators to reduce the CO_2_ emission and the carbon footprint of internal combustion engine cars that simultaneously require exact standards of performance and quality. This sector is also under increasing pressure to meet environmental and performance demands at competitive costs, leading to disruptive changes in how materials are produced, used, and managed at the end of life [35]. Biobased plastics, based on renewable resources, reduce the dependency on fossil fuels and the carbon footprint of vehicles. This test case in automotive intends to outperform state-of-the-art biobased components for interior use of the vehicle by providing a biobased nano-reinforced solution. Specifically, photocurable resins, biobased additive manufacturing, nanocomposite additive manufacturing, and Polyurethane and additive manufacturing are the topics used in screening patents in the automotive field.

The second test case is the agricultural sector, where biodegradable and compostable products for agricultural applications can substitute traditional plastic materials. This is a sector where biobased products have the potential to revolutionize modern agriculture by providing sustainable and eco-friendly alternatives to traditional chemical-based products [28]. BIOMAC experts suggest that succinic and lactic acid derived from biomass can be used as monomers in the development of completely biodegradable succinate and PLA biopolymers. Biodegradable plastic, mulch films, grow pots, and film extrusion are the topics used in screening patents in the agriculture field.

The third test case is the construction industry, where the innovative incorporation of renewable and biodegradable materials into the existing construction technology can meet the sustainability demand and provide a novel approach to replace or improve complicated construction works [36]. Materials and processes in this sector have a significant environmental impact; therefore, it is urgently required to redefine standards and procedures to meet the goal of reduced carbon emissions. Isocyanate-free Thermoplastic Polyurethane (TPU) biopolymers reinforced with biochar and Nano Fibrillated Cellulose (NFC) are among the methods for the development of biobased products such as footbridge modules with 3D printing technology. Biobased construction, nanocomposite construction, polyurethane construction, and biobased/biomass footbridge are the topics used for screening the patents in the construction field.

The fourth test case is food packaging, which has long been characterized by an enormous and indiscriminate consumption of plastics, which are quickly thrown away after use. BIOMAC experts suggest developing films that will be 100% biodegradable and compostable, with increased storage and life cycle and excellent oxygen barrier properties. In such applications, food packaging, blown film extrusion, thermoforming, nanopatterning, biodegradable/compostable PLA film, and nanoimprint lithography (NIL) self-cleaning, antibacterial surface are the topics used to search the patents in the food packaging field.

The fifth application for biobased materials is printed electronics, where connected wearable products are developed. These products are expected to have good performance and quality in preventing musculoskeletal disorders. TPU electronics, screen printing, wearable electronics, printed electronics, thermal bonding, flexible electronics, and inks are the main topics used in searching patents in the printed electronics field.

In general, these five test cases show a good future potential for biobased innovation and commercialization, as well as the increased awareness of customers of the negative impact of traditional materials, which showcases a growing market. The list of companies considered for this analysis with respective address are shown in Appendix A.

## 4. Results and Discussion

### 4.1. Patent Search Results

Table 1 illustrates the summary of patents by macro theme, excluding duplicates. From the three macro themes, biobased platform chemicals/monomers resulted in a higher number of patents, followed by biobased nanomaterials (considering a single query). However, biobased nanomaterials resulted in a higher number of patents when searches were employed using titles, abstracts, and objects of the invention. This indicates that the innovation trajectories are somewhat more towards biobased monomers and biobased nanomaterials.

In Table 1, the percentage shows the ratio of the total results obtained (before screening) to the result obtained after removing the duplicates (after screening). It shows the percentage of patents included in the analysis from the total initial search result. For example, for biobased nanomaterials, 80% of patents were considered as there were 20% duplicates. In the case of biobased platform chemical/monomers, there were many duplicates. The initial result was 1149, but after removing duplicates, the result became 621, which is 54% of the initial sum of results. This also works for the result summarized in Table 2.

Table 2 shows the number of patents retrieved for the five test cases. Despite the fact that the selected sectors have future potential for material innovation, food packaging, and construction applications, there has been a higher number of patents compared to the other three applications. This could be due to (1) the constant demand for environmentally friendly and safe products and (2) innovations in these highly regulated applications, as well as patents that could be filed to protect innovations that comply with such regulations. The automotive sector shows a lower number of patent filings. However, the automotive industry is facing significant pressure towards green transition and global competitiveness [35]), which will shape more innovations in the sector.

Summing up the total of patents retrieved for materials (1619) and test cases (1462), we obtained a final database of 2843. In a nutshell, 92% of the merging between materials and test cases was included in the sample, showing a low level of redundancy and further confirmation about the precisions of the selected queries. Table 3 and Table 4 summarized the queries employed in searching and the results obtained for the materials and test cases, respectively.

Considering the specific topics, Sugar Alcohols/polyols/diols/glycols (13%), biobased polyester (8.22%), PlA (6.08%), and nanocellulose (32.22%) are the specific topics where a greater number of patents are claimed. There may be several reasons behind this. For example, PLA is one of the most promising biobased polymers [37] due to its availability, compostability, biocompatibility, and properties close to conventional fossil-based polymers [32]. Similarly, biobased materials such as cellulose, hemicellulose, and lignin are bio-compatible and suitable for different applications. In Table 3 and Table 4, the query represents the keywords used to search for patents in an ORBIT platform. The search was limited to a title (TI), abstract (AB), claims (CLMSs), and object of invention (OBJ) as described in the methodology section.

### 4.2. Biobased Technological Trajectories

**Figure 5:**
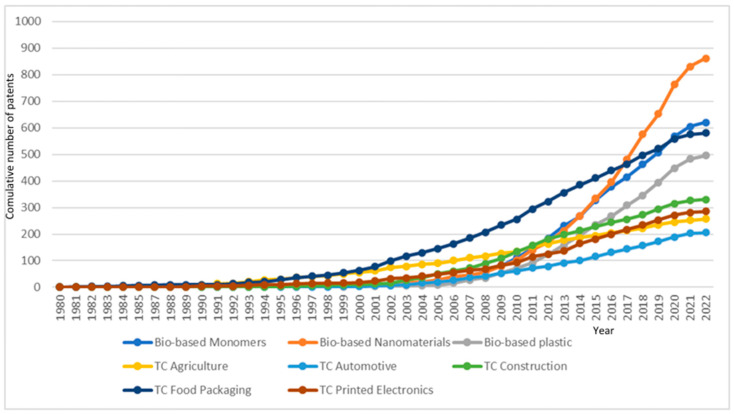
Trends in the cumulative number of biobased materials patents by macro theme.

Biobased patents record incremental growth. Biobased materials such as cellulose, hemicellulose, and lignin and their composites are not only abundant on earth but also bio-compatible and suitable for different applications, which makes them the first choice for transformative device fabrications. However, biobased nanomaterials have shown rapid growth recently. The number of patents in the selected five test cases also showed incremental growth as different innovative biobased products are being produced and delivered to the market. Despite the fact that the commercial use of biobased and biodegradable food packaging materials is still low compared to conventional materials [32], material innovation in this application shows strong improvement. Figure 5 summarizes such results.

**Figure 6:**
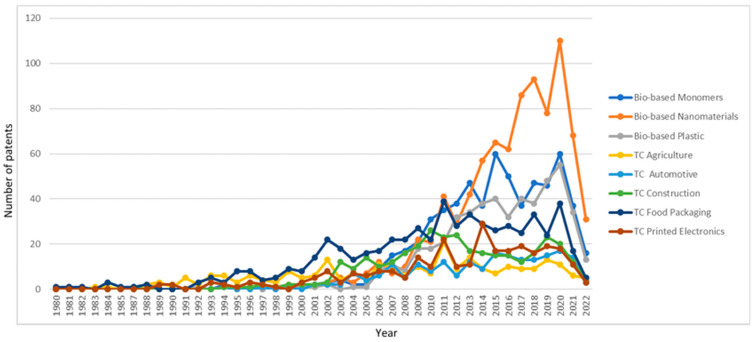
Patents by year for each macro theme.

Considering patents per year for each macro theme, biobased nanomaterial also showed a rapid increment, as shown in Figure 6. Their application in various industries such as health, agriculture, food, the textile industry, the environment and energy, and the production of materials and tools leads to several innovations in biobased nanomaterials. Biobased monomers and biobased plastics also showed increments in their patent filing. Moreover, the patents in the five test cases showed significant growth recently. This indicates that the innovations of biobased materials for the specified applications (markets) are increasing. This could be partly due to the significant regulatory pressures and the augmented customer demand for such products, which, in general, have become a competitive edge for companies. However, in the year 2022, the graph shows a decrement in all applications. This is because the ORBIT platform necessitates a certain period to collate and report comprehensive data from various repositories. This delay in data acquisition could result in the exclusion of numerous patents in the representation of the patent landscape over the years.

**Figure 7:**
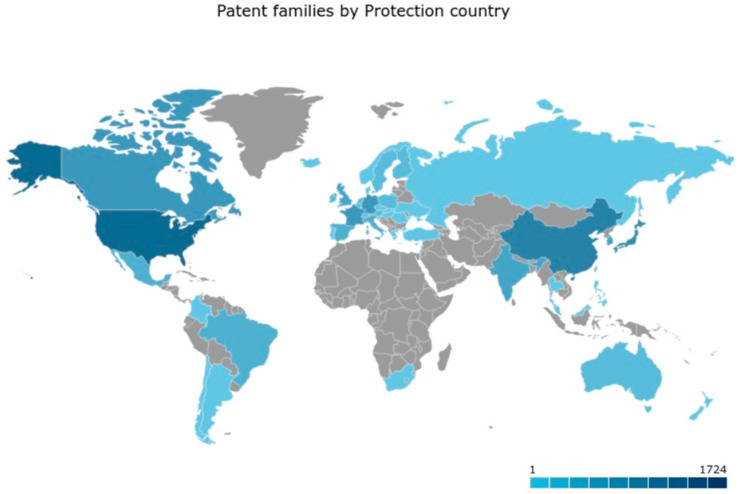
The geographical distribution of the biobased patents.

Observing the geographical distribution of players, the US and China are leading the biobased innovation landscape. Japan, Germany, and France are the followers, as shown in Figure 7. However, considering only technological advancements in biobased polymers and biobased monomers, China leads the biobased innovation significantly, followed by the US, Japan, and Germany.

**Figure 8:**
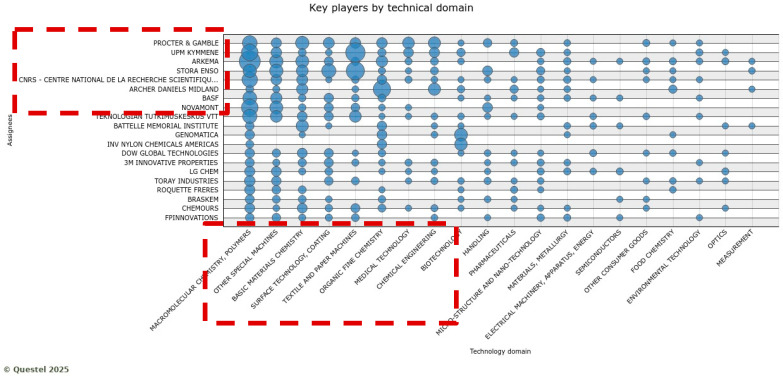
Key players in the biobased materials by technology domain where the box in red shows the companies actively engaged in bio-based innovation with respective knowledge domians (Note: the full name of assignee shown in … is CNRS-centre national de la recherche scientifiqe).

Further analysis was devoted to analyzing the characteristics of the market for biobased materials, using three indicators: technology domains, patent portfolio size, patenting period, and geography. The analysis of the technology domains, reported in Figure 8, suggests that few assignees (such as Procter and Gamble, UPM, Arkema, Stora Enso, CNRS, BASF, and Novamont) play a significant role in some specific technology domains (such as molecular chemistry, polymers, special machines, basic materials, medical technology, biotechnology, textile and paper machines, and chemical engineering), which seem to be the most attractive for the market and grant the possibility to host multiple applications. As a consequence, the market for these technologies is concentrated around these specified big players.

Another important consideration is the portfolio size. A bigger portfolio size means a significant R&D activity that spans multiple technological trajectories, de facto building high barriers for competitors to enter the market. Companies that show higher innovation capacity, market influence, and product diversification are Novamont, Arkema, BASF, and Procter and Gamble. In particular, Novamont plays a significant role in biobased innovation for agricultural and food packaging applications, where the company shows long-term patenting activity and a large patent portfolio. Similarly, Arkema plays a significant role in biobased innovation for automotive and construction applications.

**Figure 9:**
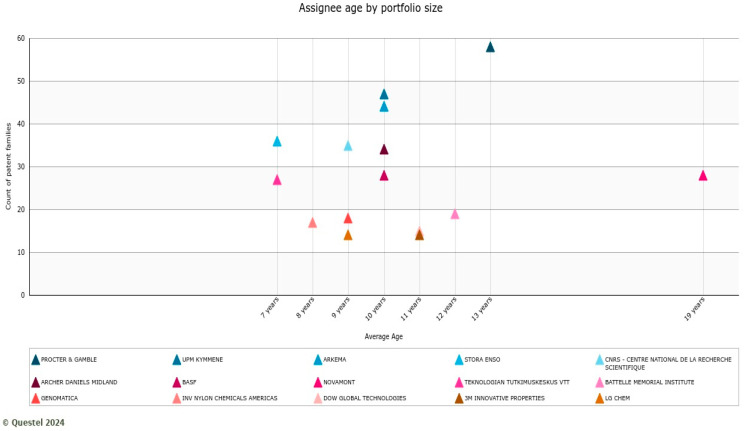
Assignee age by portfolio size.

Considering the patenting period, which gives insights into the knowledge accumulation in biobased materials innovation and is measured as the number of patents across years by assignee, Novamont has the longest patenting period with an average number of patents. Procter and Gamble also show the highest number of patents with the second average age after Novamont. These companies have maintained consistent biobased innovation, have well-established markets, and diversified products for different applications despite the upscaling and commercialization of such innovations being at early stages compared to their traditional counterparts. Figure 9 illustrates these results.

A combination of a large patent portfolio and long-term patenting experience in the field is particularly important to becoming leaders in the exploitation of technologies, and the biobased materials market structure clearly shows how it is dominated by a few specialized players. Procter and Gamble, UPM, Novamont, Arkema, and others, as shown in Figure 9, are among the top 10 players.

**Figure 10:**
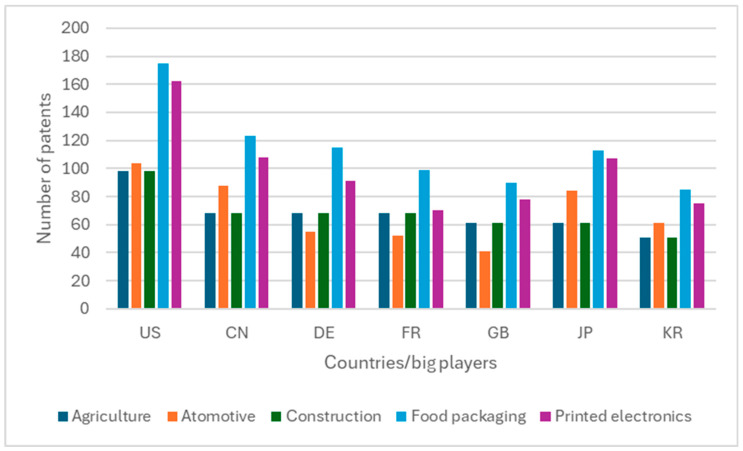
Patent families by protection country for the five test cases (United States, CN-China, DE-Germany, FR-France, GB-Great Britain, JP-Japan, KR-South Korea).

A final remark is due to the geographical distribution of the major players. The market for innovations in the five test cases (agriculture, automotive, construction, food packaging, and printed electronics) is concentrated in the US, China, Germany, France, Great Britain, Japan, and Korea, as shown in Figure 10. In these countries, the majority of the patents are in food packaging, followed by printed electronics and automotive manufacturing.

In sum, considering the technology domain by assignees, portfolio size, and geography, our results suggest that the market is heavily concentrated, with a few key players mainly located in the US, China, Germany, France, GB, and Japan. For sure, we are assisting in a clear effort to conduct R&D activities in the biobased materials, but so far, the reach of a paradigm shift from the traditional fossil-based economy towards a more sustainable economy with products of biological origin is hampered by the existence of a concentrated market that might create barriers for minor players to enter and consequently reducing the pace of the scaling up of biobased products [38]. Though biobased products are used in an increasing number of markets, their upscaling and commercialization are hampered by different factors such as high production costs, low yield, desired mechanical properties, legislation, and end-of-life options [10,13,32,35]. The future for biobased innovation is full of potential for upscaling and commercialization if an open innovation framework is adopted [16], allowing for collaborations between large and established companies and small start-up companies. This innovation strategy may speed up the penetration of biobased materials in the market and reach a relevant threshold in terms of players in the market (with increased competition, lower R&D costs, and lower prices for customers).

### 4.3. Biobased Materials Key Players: Evidence from the Test Cases

Analysis was conducted for the five test cases: agriculture, automotive, packaging, construction, and printed electronics applications. Key players and knowledge domains in each test case have been examined.

**Figure 11:**
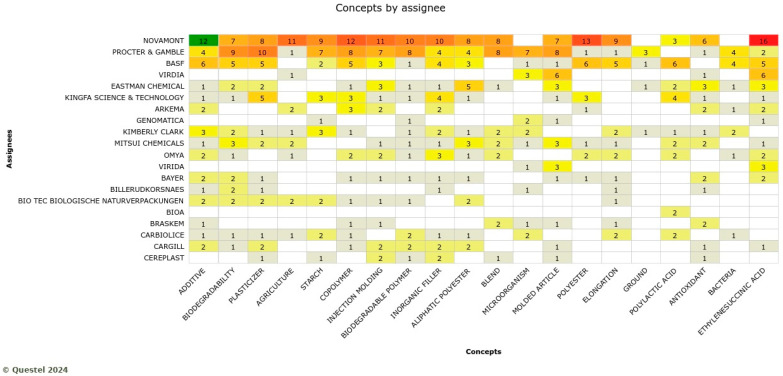
Knowledge domains by the assignee in the agricultural sector where the more red the colres indicate the larger numbers of concepts by assignees.

Novamont, Procter and Gamble, and BASF are the top three players in innovations in agricultural applications. The knowledge domains include additive, copolymer, plasticizer, injection molding, biodegradable polymer, copolymer, inorganic filler, polyester, and ethylene succinic acid, as shown in Figure 11.

**Figure 12:**
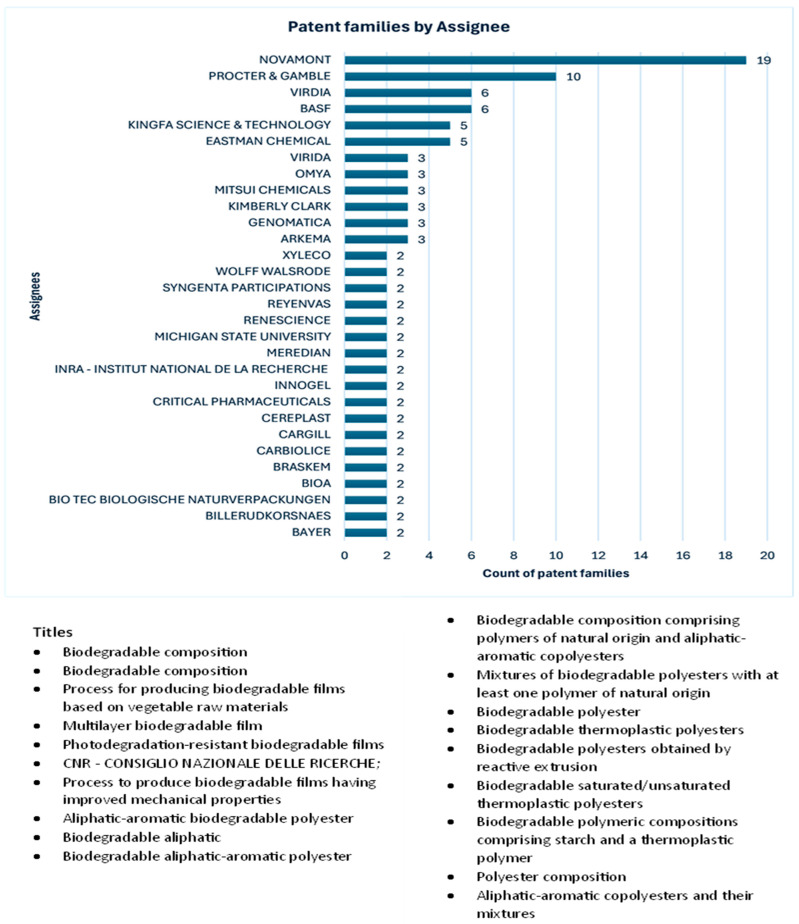
List of patents filed by Novamont for agricultural applications.

Companies such as Novamont are working on innovations for agricultural applications, such as biodegradable soil mulch films and biodegradable stackable grow pots for home gardens, plant nurseries, and commercial farms. Mulching films are used in a consolidated agronomic technique to prevent weed growth, improving soil health and fertility while preserving soil moisture. Such products are expected to solve some specific environmental problems, such as white pollution and soil erosion from the use of non-biodegradable mulch films. Biodegradable stackable grow pots are also important innovations that are extensively used in home gardens, plant nurseries, and commercial farms due to the reduced cost of thin, disposable plastic pots. This product could offer improved recyclability options (including organic recycling). Figure 12 shows the list of topics on which Novamont focuses its innovation and marketing. Novamont is working on capturing the market through biodegradable products.

**Figure 13:**
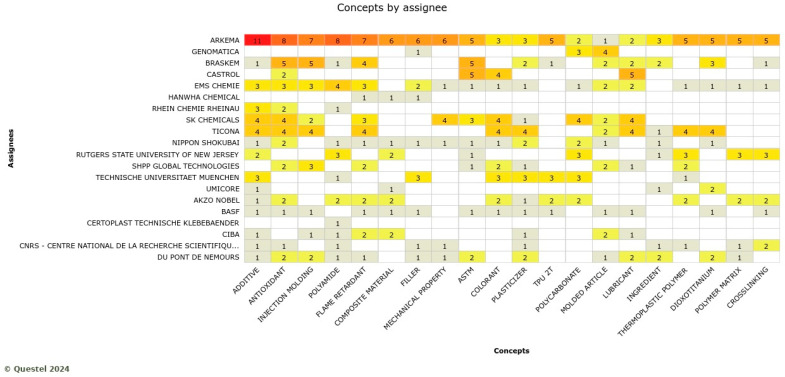
Knowledge domains by the assignee in the automotive sector where the red colors indicate the large number of concepts by assignees (Note: the full name of assignee shown in … is CNRS-centre national de la recherche scientifiqe).

For automotive, Arkema, Genomatica, and Braskem play a leading role in several knowledge domains, such as additive, antioxidant, injection molding, polyamide, and flame retardant. Sk Chemicals and Ticona are active in different subthemes (Figure 13). These innovations are for interior and exterior use for cars. For example, a sun visor is a biobased product for interior use that was recently developed by DIAD (a partner in the BIOMAC project). In pursuing this technological trajectory, companies are oriented to empower existing products with exceptional physical properties, including toughness, flexibility, and resistance to abrasion and temperature. Products will also have a unique composition with a higher biobased content.

**Figure 14:**
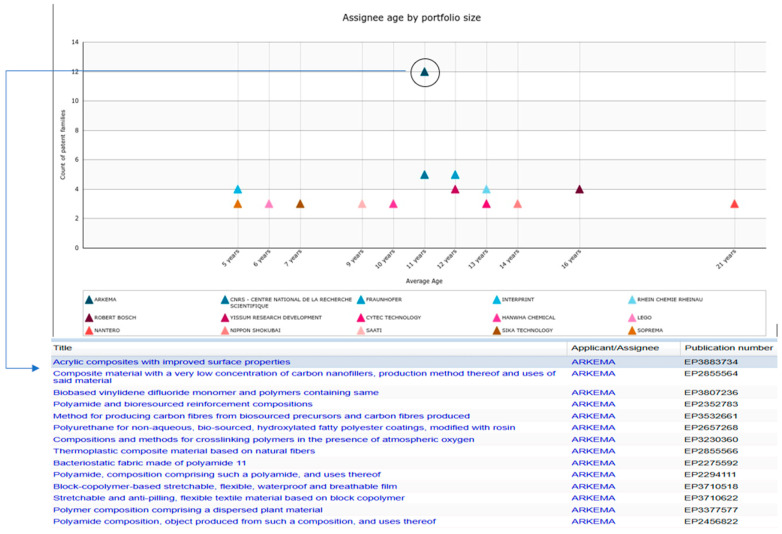
Portfolio size and list of patents filed by ARKEMA for automotive applications.

Examining the knowledge domains of ARKEMA (a leading player in automotive applications), biobased and bio-sourced composite materials are the main objectives of the innovation efforts. ARKEMA is working to capture the automotive market through bioplastic products with better mechanical properties. Figure 14 shows the portfolio size and list of ARKEMA’s patented innovations for automotive applications.

Even though many areas of the market for biobased car components are still in the testing stage [38], the uptake of electric cars and vans has increased (e.g., in Europe) significantly in recent years, which shows the market transition is already translating into new needs on the material level [35].

**Figure 15:**
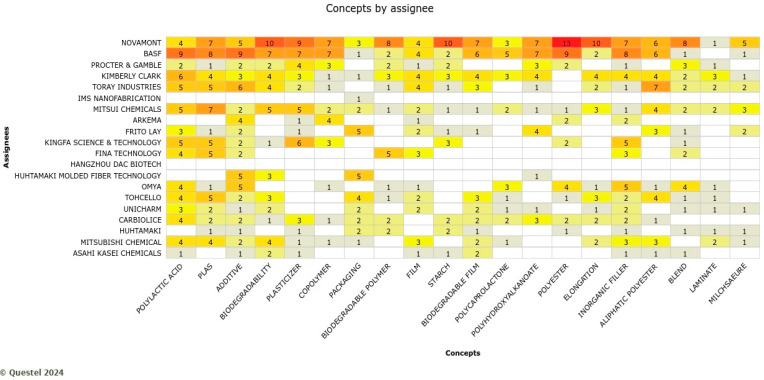
Knowledge domains by the assignee in the packaging sector where the more red colors indicate the more active companies with respective concepts.

For Food packaging, Novamont and Basf play a leading role, resulting in the assignees with the highest value of occurrence, as shown in Figure 15. Plasticizer, starch concepts, polylactic acid, biodegradability, polyester, and blend are the knowledge domains highly practiced in this application. Apart from innovations for agricultural applications, Novament is again playing a leading role in packaging materials. Such innovations focused on proposing improved biodegradability and recycling. To make this happen, some companies in this sector are working towards developing custom-configured packaging films based on biobased PLA film reinforced with bacterial nanocellulose (BNC) and nanolignin (NL) and coated with NFC coatings. Such products are expected to be fully biodegradable and compostable with excellent performance for food safety and maintenance, solve problems related to the disposal of solid waste, and ensure full circularity.

**Figure 16:**
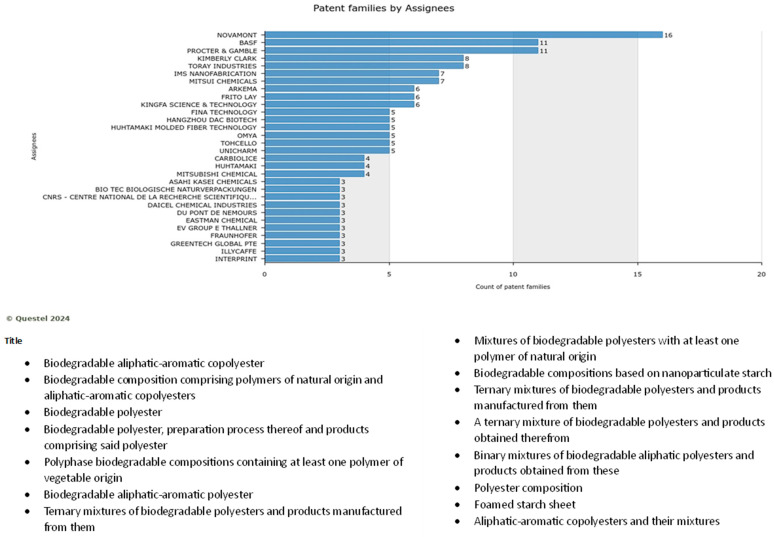
Portfolio size and list of patents filed by Novamont for food packaging applications (Note: the full name of assignee shown in … is CNRS-centre national de la recherche scientifiqe).

Novamont is also leading the research on food packaging applications and biodegradable food packaging bioplastics, as shown in Figure 16.

**Figure 17:**
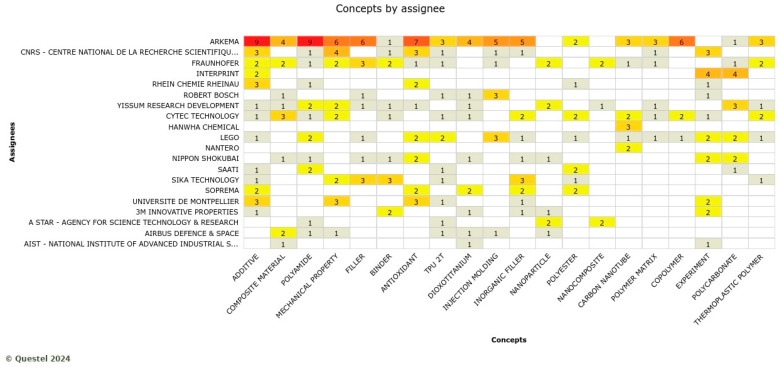
Knowledge domains by the assignee in the construction sector where the more red the colores indicate the more active companies in the corosponding concepts (Note: the full names of assignees shown in … are CNRS-centre national de la recherche scientifiqe, and AIST-national institute of advanced industrial science and technology).

Arkema confirms its multi-businesses orientation, playing a leading role also for patents retrieved within the construction applications (Figure 17). Arkema’s dominance in this application is in additive, polyamide, antioxidant, filler, and copolymer knowledge domains. These companies are developing biobased products for construction applications such as footbridges using composite materials. These products will lower lead times due to faster manufacturing (owing to the elimination of time-consuming, complex form-work fabrication), transportation, and installation time (due to the lightweight of this pedestrian bridge) as compared to a pedestrian bridge constructed using conventional materials like concrete and steel. More importantly, these products are biobased and recyclable with good mechanical properties (such as being lightweight). To realize the intricate construction mark application, the convergence between biotechnological processing and existing construction-related material synthesis or construction technologies is necessary [36].

**Figure 18:**
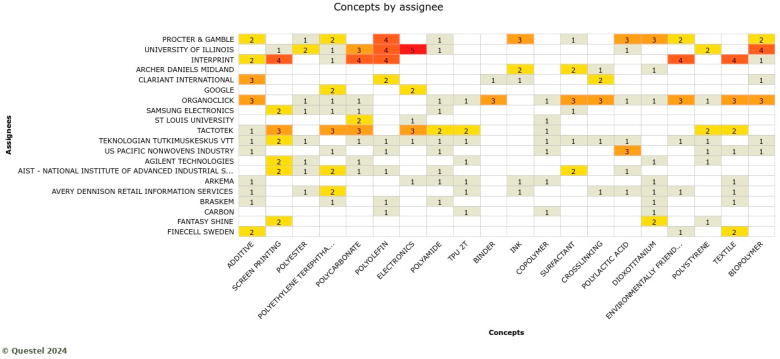
Knowledge domains by the assignee in the printed electronics sector where the more red colors indicate actively engaged companies with respective concepts (Note: the full name of assignee shown in … is AIST-national institute of advanced industrial science and technology, and in the concepts axis is environmental friendly).

In the printed electronics sector, Procter and Gamble, the University of Illinois, and Interprint play the key role. For this group, it is interesting to register two assignees who were not in the previous test cases: the University of Illinois and Interprint (Figure 18). Electronics, polyolefin, polycarbonate, screen printing, and biopolymer are relatively frequent concepts considered by key players. These companies are developing products such as a wearable sensor sleeve for the elbow to use to prevent work-related injuries due to unhealthy work standards by detecting when high strains occur and changing work standards accordingly. Such innovation may have stretchable conductive layers embedded into textiles with print processes using biobased stretchable substrates.

**Figure 19:**
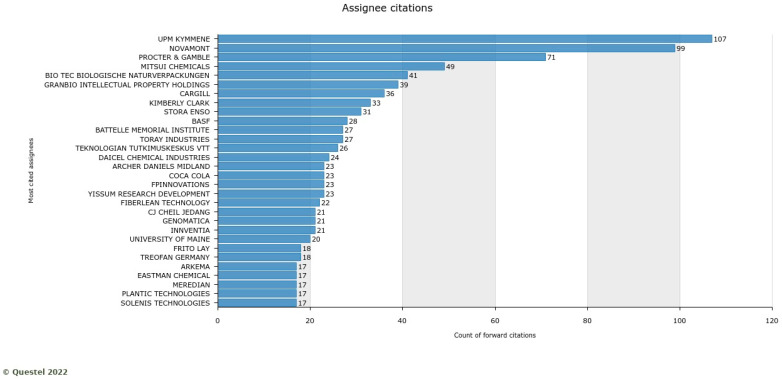
Assignees with the highest citations.

To understand the impact of assignees in knowledge production, we also report the most cited assignees. Novamont is the second largest actor with 99 citations, following UPM (Figure 19), which indicates how positively Novamont is influencing biobased innovation in agricultural and packaging applications. This also shows that they have good portfolio quality with better market potential.

In the five test cases selected for this analysis, Novamont (a partner in the BIOMAC project) is playing a leading role in agricultural and food packaging applications. Arkema is among the leading players in the market in the selected five applications. From the five markets, the printed electronics sector has players with small portfolio sizes. One reason might be embedding bioplastics with digital technologies is a new area of innovation that might attract innovations soon.

To understand the possibilities of commercializing similar technologies, we conducted a freedom-to-operate analysis following the QUESTEL guideline. The search result shows that in a global context, food packaging (36.03%) is a relatively heavily protected sector, followed by the construction sector (24.64%). However, the total number of FTO search results is high considering the broadness of the five applications, suggesting that there is a strong potential for commercializing products without infringing on others. Specifically, the FTO search result in Europe for the selected applications is low (only 699). This suggests that the biobased innovation for these applications is emerging, few active players are securing their IP, and there is a promising potential to innovate and commercialize biobased products without violating other protections.

## 5. Summary

This study provides an in-depth exploration of biobased materials innovation, focusing on key players, evolutionary trends, and patent-driven technological advancements. Leveraging the Horizon 2020 BIOMAC project framework, it investigates biobased monomers and polymers to uncover market dynamics and innovation strategies within five key sectors: automotive, agriculture, construction, food packaging, and printed electronics. China and the U.S. emerge as leaders in the biobased innovation landscape, with Europe showing strong growth potential. This research identifies leading assignees, including Novamont, Arkema, and BASF, highlighting their roles in advancing biobased materials for applications such as biodegradable packaging and automotive components. This study emphasizes the importance of collaborative R&D, open innovation frameworks, and freedom-to-operate strategies to overcome market barriers and promote the commercialization of biobased solutions. By mapping technological domains and market trends, it provides a comprehensive roadmap for advancing sustainable practices and scaling biobased innovations.

## 6. Conclusions

Knowing that between 40% and 50% of all plastic produced is single-use plastic used for product packaging [39], biobased biodegradable innovations could be good solutions to replace plastic production, reducing the environmental effect. In this regard, a growing population, urbanization, increased awareness, expanded application, and increased environmental concerns are opportunities driving demand for biobased innovations and market growth in the coming years [10]. It is, therefore, of paramount importance to detect current technological trajectories in the biobased materials field, identify what are the most promising applications, evaluate the market potential for innovative solutions, and identify the key players in the market that can possibly be included in a fruitful R&D collaboration network. In other words, it is crucial to picture the freedom-to-operate for scientists who operate at the forefront of this technology paradigm shift.

As technological space has many dimensions [26], mapping technological innovation is a challenging task, especially for biobased innovations where there are several stakeholders in the process. Despite the challenge, the innovation of biobased materials has exhibited a rapid increase over the last few years. Recently, the development of biodegradable polymers has been recognized as one of the most successful innovations in the polymer industry in addressing environmental issues [11].

This study highlights the transformative potential of biobased materials in addressing global sustainability challenges, with a particular focus on their application across key sectors such as automotive, agriculture, construction, food packaging, and printed electronics. The analysis of patent landscapes underscores the increasing innovation trajectories, particularly in biobased nanomaterials and monomers, driven by regulatory pressures, environmental concerns, and customer demand for sustainable alternatives.

To understand the biobased innovation landscape and market size concentration, this work took as a unit of analysis the patenting activity in five test cases (automotive, agriculture, construction, food packaging, and printed electronics), considering geography, knowledge domain, and portfolio size and patenting period. Geographically, the US and China are the biggest leaders in biobased innovation. However, China is becoming a leader in the innovation of biobased polymers and monomers. In the five selected test cases, the US, China, Germany, France, Great Britain, Japan, and Korea are playing a key role, and the innovation efforts of these countries are particularly high in the food packaging and printed electronics fields. The finding shows that the recent innovation trajectories are towards polymers, molecular chemistry, biotechnology, medical technologies, and textile applications. In the process, the key performing companies include Procter and Gamble, UPM, Arkema, Stora Enso, CNRS, BASF, and Novamont. We also employed analysis to understand where these biobased innovations are concentrated using the technology domain, portfolio size, and patenting period as indicators. The result showed that most innovations and markets revolve around key players such as Procter and Gamble, UPM, Arkema, Stora Enso, CNRS, BASF, and Novamont. Novamont works in the agricultural and food packaging markets, focusing on biodegradable and compostable products. Arkema, on the other hand, plays a leading role in automotive and construction markets, emphasizing biobased and bio-sourced composite products. The printed electronics market, however, is the sector with players with small portfolio sizes and patenting periods with promising future potential. This is a new market space for innovations embedding bioplastics with digital technologies (such as the Internet of Things virtual/augmented reality) for upcoming customer needs.

Despite advancements, challenges persist, including high production costs, scalability issues, and market concentration, which may hinder broader adoption. This study advocates for adopting open innovation strategies and fostering collaborations between academia, industry, and technology developers to unlock untapped potential and reduce entry barriers for emerging players. The open innovation strategy is rooted in the opportunity to access and recombine external to the company knowledge with internal resources [40], and it plays a crucial role in the development of biobased materials [16].

It is believed that to meet the requirements of the eco-conscious consumer market and enhance product longevity, sustainable solutions relying on biobased and/or biodegradable polymer systems must be viable [41]. Recently, the development of biodegradable polymers has been recognized as one of the most successful innovations in the polymer industry in addressing environmental issues [11]. The findings suggest that the future of biobased materials lies in enhancing regulatory frameworks, supporting collaborative innovation, and fostering technological diversity.

With the right regulatory frameworks in place [28], these innovative products can help support sustainable practices and promote a healthier environment for all, ensuring positive commercial impacts.

Biobased innovations and upscaling of productions are key factors in fulfilling the growing awareness and demand. Companies should change their view towards using patents as a commercialization tool rather than just the protection of innovations. Similarly, researchers can refer to the deep analysis provided in this study to identify directions for biobased innovations not already covered by patents and connect with key players to develop collaboration for innovation or research with potential patent application development. In sum, in this paper, we discuss the potential transition in the leadership of the biobased innovation landscape, the emergence of novel knowledge domains in the biobased innovation, and the market size concentration of these innovations. However, this study only considered the five sectors that were used to analyze the patent data. Performing a similar analysis and comparing the results for other sectors might be a potential future research direction.

Our research offers important implications for material scientists. First, they should prioritize research in sectors with strong market potential, such as food packaging and automotive applications, where biobased materials can offer significant environmental benefits and meet regulatory demands. Second, they should increasingly engage in partnerships with industry leaders, multiple research institutions, and policymakers in order to accelerate the development of innovative materials and reduce the time to market. Collaborative frameworks, such as those provided by the BIOMAC project, can serve as effective platforms for advancing biobased technologies. Third, given the rapid growth in biobased nanomaterials, scientists should investigate their properties and applications in emerging fields, including printed electronics and medical technologies, to address evolving market needs. Fourth, by analyzing existing patents and technological landscapes, material scientists can identify gaps in current research, avoid duplication, and focus on unique and commercially viable innovations.

This study is not without limitations. We considered only patent data related to five sectors; future research could be dedicated to performing similar analyses in other sectors and comparing the results. Further research into additional sectors and cross-industry applications will be critical to driving the next wave of sustainable material innovations.

## Figures and Tables

**Table 1:** Summary patents were retrieved for materials, excluding duplicates (source: authors’ elaboration based on ORBIT data).

Themes	Search by Title, Abstract, and Claims and Object of the Invention (Excluding Duplicates)	Total Number by the Sum of Single Query	Result After Screening (%)
Biobased platform chemicals/monomers	621	1149	0.54
Biobased plastic	496	728	0.68
Biobased nanomaterials	862	1030	0.84
Total	1619	2907	0.56

**Table 2:** Summary of patents retrieved for test case excluding duplicates (source: authors’ elaboration based on ORBIT data).

	Search by Title, Abstract, and Claims and Object of the Invention (Excluding Duplicates)	Total Number by the Sum of Single Query	Result After Screening (%)
Food packaging	581	596	0.97
Construction	331	363	0.91
Printed electronics	285	304	0.94
Agriculture	257	279	0.92
Automotive	206	235	0.88
	1462	1777	0.82

**Table 3:** Results for the selected queries for materials (source: authors’ elaboration based on ORBIT data).

Macro Theme	Topic	Query	Number of Patent _World	Number of Patent _US	Number of Patent _EPO
Biobased platform chemicals/monomers	Succinic acid	(((SUCCINIC ACID))/TI/AB/CLMS/OBJAND ((BIO_BASED))/TI/AB/CLMS/OBJ)	414	151	105
	Lactic acid	(((LACTIC ACID))/TI/AB/CLMS/OBJ AND ((BIO_BASED))/TI/AB/CLMS/OBJ)	413	189	151
	Sugar Alcohols/polyols/ diols/glycols	((((Sugar alcohol?) OR (polyol?)) OR (diol?) OR (glycol?))/TI/AB/CLMS/OBJ AND ((BIO_BASED))/TI/AB/CLMS/OBJ)	2224	706	511
	Sorbitol	(((sorbitol))/TI/AB/CLMS/OBJ AND ((BIO_BASED))/TI/AB/CLMS/OBJ)	363	115	98
	Ethylene glycol	(((ethylene glycol))/TI/AB/CLMS/OBJ AND ((BIO_BASED))/TI/AB/CLMS/OBJ)	740	212	156
	Propylene glycol/1,2 propanediol/1,3 propanediol	((((propylene glycol?) or (1,2 propanediol?) or (1,3 propanediol?)))/TI/AB/CLMS/OBJ AND ((bio_based))/TI/AB/CLMS/OBJ)	537	175	128
Biobased plastic	Polylactic acid	(((polylactic acid) OR (PLA))/TI/AB/CLMS/OBJ AND ((BIO_BASED))/TI/AB/CLMS/OBJ)	1040	268	226
	Succinate polyester (to refine biobased polyester)	((((succinate) and (polyester)))/TI/AB/CLMS/OBJ AND ((BIO_BASED))/TI/AB/CLMS/OBJ)	165	72	57
	Lactate polyester (con biobased, variate di biobased polyester)	((((lactate) and (polyester)))/TI/AB/CLMS/OBJ AND ((BIO_BASED))/TI/AB/CLMS/OBJ)	19	13	9
	Biobased polyester	(((polyester))/TI/AB/CLMS/OBJ AND ((BIO_BASED))/TI/AB/CLMS/OBJ)	1406	496	367
	Polyurethane resin	(((polyurethane resin?))/TI/AB/CLMS/OBJ AND ((BIO_BASED))/TI/AB/CLMS/OBJ)	138	26	24
	TPU	(((TPU) or (thermoplastic polyurethane))/TI/AB/CLMS/OBJ AND ((bio_based))/TI/AB/CLMS/OBJ)	131	46	45
Biobased nanomaterials	Conductive biochar	(((biochar))/TI/AB/CLMS/OBJ AND ((conductive))/TI/AB/CLMS/OBJ)	174	27	16
	Biochar and copper and silver	(((biochar))/TI/AB/CLMS/OBJ AND ((copper) or (silver))/TI/AB/CLMS/OBJ)	721	53	22
	Biobased lignin	(((lignin))/TI/AB/CLMS/OBJ AND ((BIO_BASED))/TI/AB/CLMS/OBJ)	521	161	131
	Nanolignin	((Nanolignin) or (nano_lignin))/TI/AB/CLMS/OBJ)	125	3	2
	Nanocellulose	(((Nanocellulose) or (nano_cellulose))/TI/AB/CLMS/OBJ)	5512	541	386
	Cellulose nanocrystals	(((cellulose)/TI/AB/CLMS/OBJ AND (nano_crystals)/TI/AB/CLMS/OBJ)	1734	447	288
	Nanofibrillated cellulose or cellular nanofiber	(((nanofibrillated cellulose) or (cellular nanofiber))/TI/AB/CLMS/OBJ)	270	154	132
	Bacterial nanocellulose	(((nano_cellulose)/TI/AB/CLMS/OBJ AND (bacterial)/TI/AB/CLMS/OBJ)	454	71	53
Total			17,101	3926	2907

**Table 4:** Results for the selected queries for test cases (source: Authors’ elaboration based on ORBIT data).

Macro Theme	Topic	Query	Number of Patent _World	Number of Patent _US	Number of Patent _EPO
Automotive	Photocurable resins	(((photocurable S resin?))/KEYW/TI/AB AND ((bio_based))/KEYW/TI/AB)	20	1	1
	Biobased additive manufacturing	(((additive manufacturing or automotive))/TI/AB/CLMS/OBJ AND ((bio_based))/TI/AB/CLMS/OBJ)	195	105	100
	Nanocomposite additive manufacturing	(((nano S composite))/TI/AB/CLMS/OBJ AND (((automotive or additive manufacturing)))/TI/AB/CLMS/OBJ)	988	136	110
	Polyurethane and additive manufacturing	(((polyurethane))/TI/AB/CLMS/OBJ AND ((automotive or additive manufacturing))/TI/AB/CLMS/OBJ AND ((bio_based))/TI/AB/CLMS/OBJ)	39	26	24
Food packaging	Food packaging	(((food S packag+))/TI/AB/CLMS/OBJ AND ((bio_based))/TI/AB/CLMS/OBJ)	199	76	71
	Blown film extrusion	((((bio_based or bio or bio_degradable)))/TI/AB/CLMS/OBJ AND ((blown film extrusion))/TI/AB/CLMS/OBJ)	52	31	28
	Thermoforming	(((thermoforming))/TI/AB/CLMS/OBJ AND ((bio_based))/TI/AB/CLMS/OBJ)	49	30	32
	Nanopatterning	(((nanopatterning))/TI/AB/CLMS/OBJ)	212	110	45
	Biodegradable/compostable PLA film	(((compostable or bio_degradable))/TI/AB/CLMS/OBJ AND ((PLA or polylactic acid) S (film?))/TI/AB/CLMS/OBJ)	2555	414	360
	Nanoimprint lithography (NIL) self-cleaning, antibacterial surface	(((Nanoimprint S lithography) or (NIL))/TI/AB/CLMS/OBJ AND (((self-cleaning or antibacterial) and (surface)))/TI/AB/CLMS/OBJ)	102	74	60
Agriculture	Biodegradable plastic	(((agriculture or agricultural))/TI/AB/CLMS/OBJ AND ((plastic?))/TI/AB/CLMS/OBJ AND ((bio_based or bio_degradable))/TI/AB/CLMS/OBJ)	993	203	176
	Mulch films	(((mulch film))/TI/AB/CLMS/OBJ AND ((bio_based or bio_degradable))/TI/AB/CLMS/OBJ)	416	47	45
	Grow pots	(((bio_based or bio_degradable or bio))/TI/AB/CLMS/OBJ AND ((grow pot?))/TI/AB/CLMS/OBJ)	2	0	0
	Film extrusion	(((bio_based or bio or bio_degradable))/TI/AB/CLMS/OBJ AND ((film extrusion))/TI/AB/CLMS/OBJ)	115	59	58
Construction	Biobased construction	(((bio_based))/TI/AB/CLMS/OBJ AND ((construction?))/TI/AB/CLMS/OBJ)	367	135	145
	Nanocomposite construction	(((nano S composite))/TI/AB/CLMS/OBJ AND ((construction?)/TI/AB/CLMS/OBJ)	3096	268	189
	Polyurethane construction	(((polyurethane))/TI/AB/CLMS/OBJ AND ((construction?))/TI/AB/CLMS/OBJ AND ((bio_based))/TI/AB/CLMS/OBJ)	346	38	29
	Biobased/biomass footbridge	((bio_based or bio_mass)/TI/AB/CLMS/OBJ AND (foot_bridge)/TI/AB/CLMS/OBJ)	0	0	0
Printed electronics	TPU electronics	((((TPU) or (thermoplastic polyurethane)))/TI/AB/CLMS/OBJ AND ((electronics))/TI/AB/CLMS/OBJ AND ((bio_based))/TI/AB/CLMS/OBJ)	41	28	18
	Screen printing	(((screen printing))/TI/AB/CLMS/OBJ AND ((bio_based) or (bio) or (biodegradable))/TI/AB/CLMS/OBJ)	384	124	90
	Wearable electronics	(((wearable S electronics))/TI/AB/CLMS/OBJ AND ((bio_based) or (bio) or (biodegradable))/TI/AB/CLMS/OBJ)	55	34	20
	Printed electronics	(((printed S electronics))/TI/AB/CLMS/OBJ AND (((bio_based) or (bio) or (biodegradable)))/TI/AB/CLMS/OBJ)	50	24	19
	Thermal bonding	(((thermal bonding))/TI/AB/CLMS/OBJ AND (((bio_based) or (bio) or (biodegradable)))/TI/AB/CLMS/OBJ)	219	83	68
	Flexible electronics	(((flexible S electronics))/TI/AB/CLMS/OBJ AND ((bio_based) or (bio) or (biodegradable))/TI/AB/CLMS/OBJ)	89	47	25
	Inks	(((INK?))/TI/AB/CLMS/OBJ AND ((bio_BASED))/TI/AB/CLMS/OBJ)	210	93	64
Total			10,794	2186	1777

## Data Availability

Data are contained within the article.

## Appendix A

**Table A1:** List of companies considered when analyzing the test cases.

	End-User	Test Case	Website
1	Novamont S.p.A	Agriculture	https://www.novamont.com/
2	DIAD Group Srl	Automotive	https://diadgroup.com/
3	Eversia	Food packaging	https://eversia.es/
4	Acciona	Construction	https://www.acciona.com/
5	Precure	Printed electronics	https://precure.dk/
